# *In vitro* activities of cellulase and ceftazidime, alone and in combination against *Pseudomonas aeruginosa* biofilms

**DOI:** 10.1186/s12866-021-02411-y

**Published:** 2021-12-16

**Authors:** Esmat Kamali, Ailar Jamali, Ahdieh Izanloo, Abdollah Ardebili

**Affiliations:** 1 Infectious Diseases Research Center, Golestan University of Medical Sciences, P.O. box: 4934174515, Gorgan, Iran; 2 Department of Microbiology, Faculty of Medicine, Golestan University of Medical Sciences, Gorgan, Iran; 3 Department of Biology, Faculty of Sciences, Golestan University, Gorgan, Iran

**Keywords:** Biofilm inhibition, Ceftazidime, Cellulase, MBEC, *Pseudomonas aeruginosa*

## Abstract

**Background:**

Biofilms are a main pathogenicity feature of *Pseudomonas aeruginosa* and has a significant role in antibiotic resistance and persistent infections in humans. We investigated the *in vitro* activities of antibiotic ceftazidime and enzyme cellulase, either alone or in combination against biofilms of *P. aeruginosa*.

**Results:**

Both ceftazidime and cellulase significantly decreased biofilm formation in all strains in a dose-dependent manner. Combination of enzyme at concentrations of 1.25, 2.5, 5, and 10 U/mL tested with 1/16× MIC of antibiotic led to a significant reduction in biofilm biomass. Cellulase showed a significant detachment effect on biofilms at three concentrations of 10 U/mL, 5 U/mL, and 2.5 U/mL. The MIC, MBC, and MBEC values of ceftazidime were 2 to 4 µg/mL, 4 to 8 µg/mL, and 2048 to 8192 µg/mL. When combined with cellulase, the MBECs of antibiotic showed a significant decrease from 32- to 128-fold.

**Conclusions:**

Combination of the ceftazidime and the cellulase had significant anti-biofilm effects, including inhibition of biofilm formation and biofilm eradication in *P. aeruginosa*. These data suggest that glycoside hydrolase therapy as a novel strategy has the potential to enhance the efficacy of antibiotics and helps to resolve biofilm-associated wound infections caused by this pathogen.

**Supplementary Information:**

The online version contains supplementary material available at 10.1186/s12866-021-02411-y.

## Background

Biofilms are communities of microorganisms protected by a self-synthesized layer of complex polymers, called the extracellular polymeric substances (EPSs), represented mainly by proteins (>2%), polysaccharides (1–2%), and other constituents, such as extracellular DNA (eDNA) (<1%), RNA (<1%), and bound and free ions [1, 2]. Biofilms may form on biotic or abiotic surfaces and be prevalent in natural, industrial, and hospital settings. The microbial cells growing in a biofilm are physiologically distinct from planktonic cells of the same organism, which, by contrast, are single-cells that may float or swim in a liquid medium [3].

The biofilm mediates bacterial stability and protects them from environmental conditions, such as pH, UV damage, hydrogen peroxide, metal, and antibiotic pressure [4]. Likewise, the biofilm’s exopolysaccharide component acts as a barrier against the host immune response towards bacterial infection, including phagocytosis [5]. It has also been known that biofilm-forming bacteria are up to 1000-times more resistant to antimicrobial agents than planktonic cells, partially due to the impaired penetration [6]. Multiple mechanisms of biofilm formation and its architectural features, including decreased transportation and diffusion of antimicrobial agents into the extracellular matrix supported by glycocalyx matrix, heterogeneity in metabolism and growth rate, increased activity of multidrug efflux pumps, the formation of antimicrobial tolerant-persisters and slow-growing cells, genetic adaptation, stress responses, and quorum-sensing systems confer the increased resistance in biofilm-forming bacteria [6, 7].

According to the National Institutes of Health (NIH) agency, bacterial biofilms are involved in 65% of microbial diseases and more than 80% of chronic infections [8]. One bacterium that is particularly notorious for forming biofilms is *Pseudomonas aeruginosa* [9–11]. *P. aeruginosa* is a major nosocomial pathogen associated with a wide variety of acute and chronic infections, such as soft tissue infections, urinary tract infections, bacteremia, pneumonia, diabetic foot, otitis, and keratitis [12]. Burn wound infections are one of the most important complications following the burn injuries and may be associated with increased morbidity and mortality [13, 14]. The ability of *P. aeruginosa* to form biofilms is considered one of the main pathogenicity features of this organism in causing persistent infections in such cases, resulting in delayed healing for 2 to 4 weeks [15, 16].

Given the intrinsic resistance of *P. aeruginosa* biofilm to antimicrobial therapy, researchers are trying to develop new strategies that explicitly target established biofilms, hoping that it can significantly improve clinical practice [6]. An alternate anti-biofilm approach is the use of therapeutic enzymes that degrade the biofilm matrix. An enzyme complex (amylase, cellulase, and protease) was found that was able to degrade the biofilms of different bacteria, including *Escherichia coli* (85.5%), *Salmonella enterica* (79.72%), *P. aeruginosa* (88.76%), and *Staphyloccus aureus* (87.42%) [17]. Glycoside hydrolases (also called glycosidases) hydrolyze glycosidic bonds in complex carbohydrates [18]. Cellulase is a type of glycoside hydrolase that acts specifically by breaking down the β-1,4 linkages in polysaccharides, such as cellulose, an exopolysaccharide commonly found in the biofilm of several bacteria, including *E. coli*, *Salmonella*, *Citrobacter*, *Enterobacter*, and *Pseudomonas* as well as *Agrobacterium tumefaciens* [19]. As exopolysaccharides represent a substantial component of many bacterial biofilms and contribute to the structural stability of the EPS [20], their disruption is essential for dispersing bacteria into a planktonic state. This would increase the host immune system’s availability and administered antimicrobial compounds/antibiotics to infected cells. So, it is thought that hydrolyzing the glycosidic linkages that hold exopolysaccharides together will degrade the EPS. It has been shown that cellulase inhibits the growth of biofilms produced by *Burkholderia cepacia* and *P. aeruginosa* on various abiotic surfaces that are commonly used in medical devices [21, 22].

In this study, we aimed to determine the *in vitro* activities of anti-pseudomonal antibiotic, ceftazidime, and glycoside hydrolase enzyme, cellulase, alone or in combination against biofilms of standard and clinical isolates of *P. aeruginosa*.

## Methods

### Chemicals

Ceftazidime hydrate (CAS# 120618-65-7) and fungal cellulase (from *Aspergillus niger*) (CAS# 9012-54-8) were purchased from Sigma-Aldrich (St Louis, MO, USA). Ceftazidime stock solution was prepared by dissolving the lyophilized powder in sodium carbonate buffer. Cellulase stock solution was prepared by dissolving the dry powder in sodium acetate buffer (50 mM, pH 5) at 65 °C for 5 min. The heat-inactivated enzyme was obtained by heating the enzyme solution at 95 °C for 20 min. All stock solutions were stored at −80 °C freezer for up to 6 months.

### Culture media

Mueller–Hinton broth (MHB) was used for the determination of minimum inhibitory concentration (MIC) values and the microbroth chequerboard technique, tryptic soy broth supplemented with 1% glucose (TSB-glucose) used for biofilm production and inhibition assays, and tryptic soy agar was used for determination of the minimum bactericidal concentration (MBC) were obtained from Merck (Merck KGaA, Darmstadt, Germany).

### Bacterial strains

The bacterial strains used in all experiments were as follows: *P. aeruginosa* wild-type strain PAO1, a laboratory reference strain originally isolated in 1954 from an infected burn/wound of a patient in Melbourne, Australia (American Type Culture Collection ATCC 15,692) and two clinical isolates of *P. aeruginosa* recovered from the patients with burn wound infections that were identified by standard microbiological and biochemical methods from a previous study [9].

### Antibacterial activity assays

The MIC of ceftazidime was determined by the microbroth dilution method in 96-well polystyrene microplates (JET Biofil, Guangzhou, China) according to the Clinical and Laboratory Standards Institute (CLSI) guideline [23]. The MIC was defined as the lowest concentration of antibiotic that completely inhibited visible growth. The MBC value was determined at the end of the incubation period by removing 10 µL aliquots of all wells with no visible growth and dispensed in tryptic soy agar. Grown colonies were counted after 48 h incubation at 37 °C. The MBC was established as the lowest concentration of antibiotic that killed at least 99.9% of the initial inoculums.

The bactericidal activity of cellulase on planktonic cells of *P. aeruginosa* was investigated. Briefly, the overnight cultures were treated with two-fold serial dilutions of enzyme ranging from 0.09 U/mL to 50 U/mL in the wells of polystyrene microplate as described for the MIC determination. A well containing bacteria exposed with heat-inactivated enzyme was used as control. After 24 h incubation at 37 °C, the effect of the enzyme on the bacterial growth was determined by measuring the optical density (OD) of each culture in wells at 570 nm.

### Biofilm formation assay

Biofilm formation was assessed by the colorimetric microtiter plate assay as described previously [9]. An overnight culture of *P. aeruginosa* was adjusted to the turbidity of a 0.5 McFarland standard and then was diluted 1:100 in TSB-glucose, yielding a final concentration of about 1 × 10^6^ CFU/200 µL. This suspension was added to each well of a sterile 96-well polystyrene microplate and incubated at 37 °C for 24 h. Then, the supernatant was carefully aspirated from the wells followed by washing three times with 200 µL sterile phosphate-buffered saline (PBS, pH 7.3). Adherent biofilm in each well was fixed by 99% methanol for 15 min, the solutions were removed, and the plate was allowed to dry. Wells were stained with 200 µL of 0.1% crystal violet (CV) (Sigma Chemical Co., St Louis, MO, USA) for 5 min at room temperature, rinsed by water, and then allowed to dry. The stain was dissolved with 200 µL of 95% ethanol solution for 30 min. The OD of each well was determined at 595 nm in a microtiter plate reader (BioTek, Bad Friedrichshall, Germany). All experiments were evaluated in triplicate. A cut-off value (ODc) as three standard deviations (SD) above the mean OD of the negative control was established: ODc = average OD of negative control + (3 × SD of negative control). The isolates were divided into four categories: non-biofilm producer (OD < ODc), weak-biofilm producer (ODc < OD < 2 × ODc), moderate-biofilm producer (2 × ODc < OD < 4 × ODc), and strong-biofilm producer (4 × ODc < OD).

### Biofilm attachment assay

Biofilm attachment assays were performed using a previously described method with some modifications [24]. The overnight cultures were diluted 1:100 to give 1 × 10^6^ CFU/200 µL in TSB-glucose, and standard and clinical *P. aeruginosa* strains were added to each well of a 96-well microtitre plate with 1× and 1/2× MIC concentrations of ceftazidime or 1.25, 2.5, 5, and 10 U/mL of cellulase. The plates were incubated for 1, 2, or 4 h at 37 °C. The positive controls were *P. aeruginosa* strains in TSB-glucose without antibiotic or enzyme, and negative controls were medium with neither bacteria nor antimicrobial agents. After incubation, the wells were washed three times with PBS, and biofilm biomass was assessed by CV staining as described above.

### Inhibition of biofilm formation

Bacterial strains were prepared at a concentration of 1 × 10^6^ CFU/200 µL in TSB-glucose were added to each well of a microtitre plate with 1×, 1/2×, 1/4×, 1/8×, and 1/16× MIC concentrations of ceftazidime, 1.25, 2.5, 5, and 10 U/mL of cellulase, and combination. Wells inoculated with bacterial strains without antibiotic or enzyme were set up as positive controls. At the end of the 24-hour incubation period, wells were rinsed three times with PBS, and biofilm biomass was assessed by CV staining. The results expressed as the percentage biofilm biomass reduction compared with wells without antibiotic or enzyme.

### Biofilm treatment assay by the enzyme

The efficacy of cellulase in affecting biofilm detachment was determined. Briefly, the established biofilms in a microtiter plate were washed three times with PBS and exposed to different enzyme concentrations (range 0.01 to 10 U/mL). TSB-glucose without enzyme or bacteria was used as negative control, and biofilm with no enzyme treatment was considered positive control. A well containing bacteria treated with heat-inactivated (HI) enzyme was also used as control. After 1-h incubation at 37 °C, well contents were removed and washed thrice with PBS. Biofilm in each well was stained using the CV assay, and OD was determined at 595 nm.

### Minimum biofilm eradication concentration (MBEC) assay

Antimicrobial susceptibilities of standard and clinical *P. aeruginosa* biofilms were evaluated according to the method of Naparstek et al. [25] with few modifications. The mature biofilms in a 96-well microtiter plate were washed three times with PBS to remove planktonic bacteria. Serial 2-fold dilutions of ceftazidime ranging from 64 to 32,768 µg/mL were prepared in cation-adjusted Mueller-Hinton broth (CAMHB). A 200 µL sample of each concentration was added to a corresponding well, and plates were incubated overnight at 37 °C. The wells with established biofilm and CAMHB medium, without antibiotic and enzyme treatment were considered positive and negative controls, respectively. After the incubation, plates were washed three times with sterile PBS to remove residual antibiotic, and 200 µL of fresh CAMHB was placed in each well for further 24 h incubation at 37 °C. This allows the biofilm bacteria that survived the antibiotic exposure to grow in the absence of antibiotic and produce detectable turbidity. Subsequently, the OD of each well was determined at 595 nm. The lowest antibiotic concentration that prevented bacterial regrowth from the treated biofilm was defined as the MBEC value.

### The effect of cellulase on ceftazidime MBEC

The combined effect of cellulase and ceftazidime on *P*. *aeruginosa* biofilms was determined as described previously [26, 27]. Briefly, bacterial biofilms were exposed to 2-fold diluted ceftazidime concentrations (range 2 to 1024 µg/mL) and various concentrations of cellulase (1.25, 2.5, 5, and 10 U/mL). Following 24-h incubation, the well content was removed and washed three times with PBS, and then fresh CAMHB was added to each well for further overnight incubation. The MBEC of ceftazidime for biofilm cultures was as described above in “MBEC assay”.

### Statistical analysis

All experiments were performed in three independent assays. The results were expressed as means ± standard deviations of three independent experiments for the biofilm attachment and inhibition of biofilm formation assays. A one-way analysis of variance (ANOVA) followed by Tukey-Kramer multiple comparison tests was used to evaluate differences between groups’ mean values. A *P*-value < 0.05 was considered statistically significant. All tests were performed using GraphPad statistical software (GraphPad Software, San Diego, CA, USA).

## Results

### Susceptibility

In general, *P. aeruginosa* PAO1 strain and two clinical isolates presented low MICs to ceftazidime, in the range of the susceptible category (2-4 µg/mL) (Table 1). The MBC values were also two-fold greater than those of the MIC values.

**Table 1:** *In vitro* antibacterial and anti-biofilm activities of ceftazidime against *P. aeruginosa* strains

^a^Bacterial strain	MIC (µg/mL)	MBC (µg/mL)	MBEC (µg/mL)	Fold change in MBEC/MIC ratio	Fold change in MBEC/MBC ratio
PAO1	2	4	2048	1024	512
PA1	4	8	4096	1024	512
PA2	2	4	8192	4096	2048

*MBEC* Minimum biofilm eradication concentration, *MIC* Minimum inhibitory concentration, *MBC* Minimum bactericidal concentration

^a^ PAO1 is a laboratory reference strain of *P. aeruginosa* and PA1 and PA2 are two clinical isolates of *P. aeruginosa* that were recovered from the patients with burn wound infections

The bactericidal activity of cellulase on *P. aeruginosa* planktonic cells was investigated. The enzyme had no bactericidal effect, and bacteria grew after treatment with all concentrations (0.09-50 U/mL) of this enzyme (*P* > 0.05).

### Biofilm formation

The results of biofilm formation assay performed on three *P. aeruginosa* strains showed strong-mass biofilms (OD_595_ range 2.55–3.28) (*P* > 0.05). The isolate PA1 was found to produce more biofilm than the standard strain PAO1 or the isolate PA2.

### Biofilm attachment

**Fig. 1:**
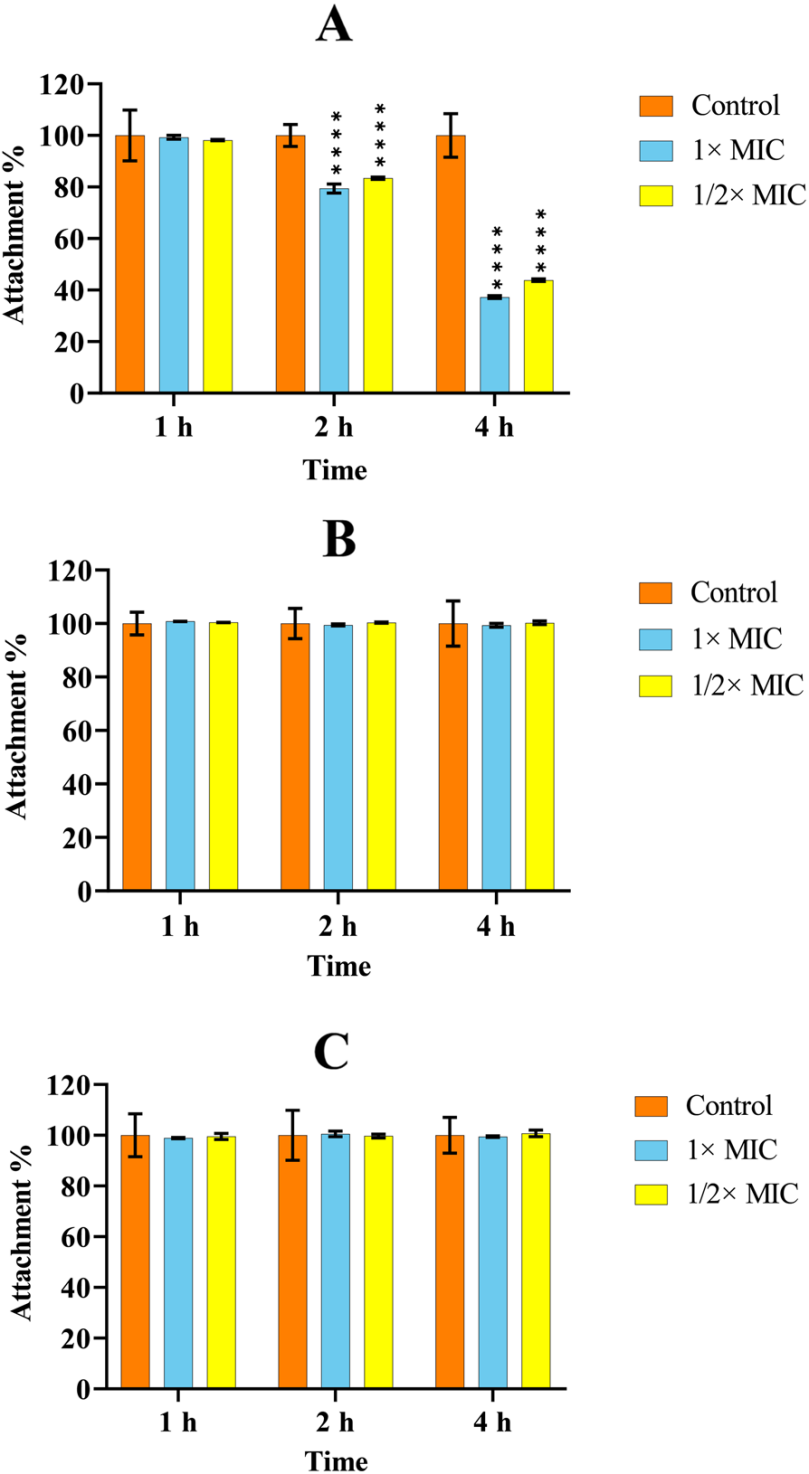
The results of the *P. aeruginosa* attachment to the surface of microplate wells containing 1× MIC and 1/2× MIC concentrations of ceftazidime in PAO1 (**A**), PA1 (**B**), and PA2 (**C**). The plates were incubated for 1, 2, or 4 h at 37 °C. Data were normalized to the mean value of the control, which was set at 100%. The error bars indicate the standard deviations between bacteria. Results were expressed as percentage of biofilm formed with respect to control. The statistical significance of the data was determined by an analysis of variance (ANOVA) test followed by the Tukey-Kramer multiple comparison test. Significance was accepted when the *P*-value was < 0.05 (****P < 0.0001). MIC: Minimum inhibitory concentration

Figure 1 shows the effects of ceftazidime at MIC, and 1/2× MIC concentrations on the adherence of *P. aeruginosa* strains to the wells of microtitre plate after 1, 2, or 4 h incubation at 37 °C. In two *P. aeruginosa* clinical isolates PA1 and PA2, none of the concentrations used inhibited biofilm attachment for each incubation period (Fig. 1B, C), while antibiotic achieved this inhibition in PAO1 strain in a concentration- and time-dependent manner; the MIC and 1/2× MIC concentrations inhibited 64% and 57% of attachment, respectively, after four h of incubation (Fig. 1 A). Besides, cellulase did not affect the attachment of *P. aeruginosa* at concentrations of 1.25, 2.5, 5, and 10 U/mL tested (Data are not shown).

### Inhibition of biofilm formation

**Fig. 2:**
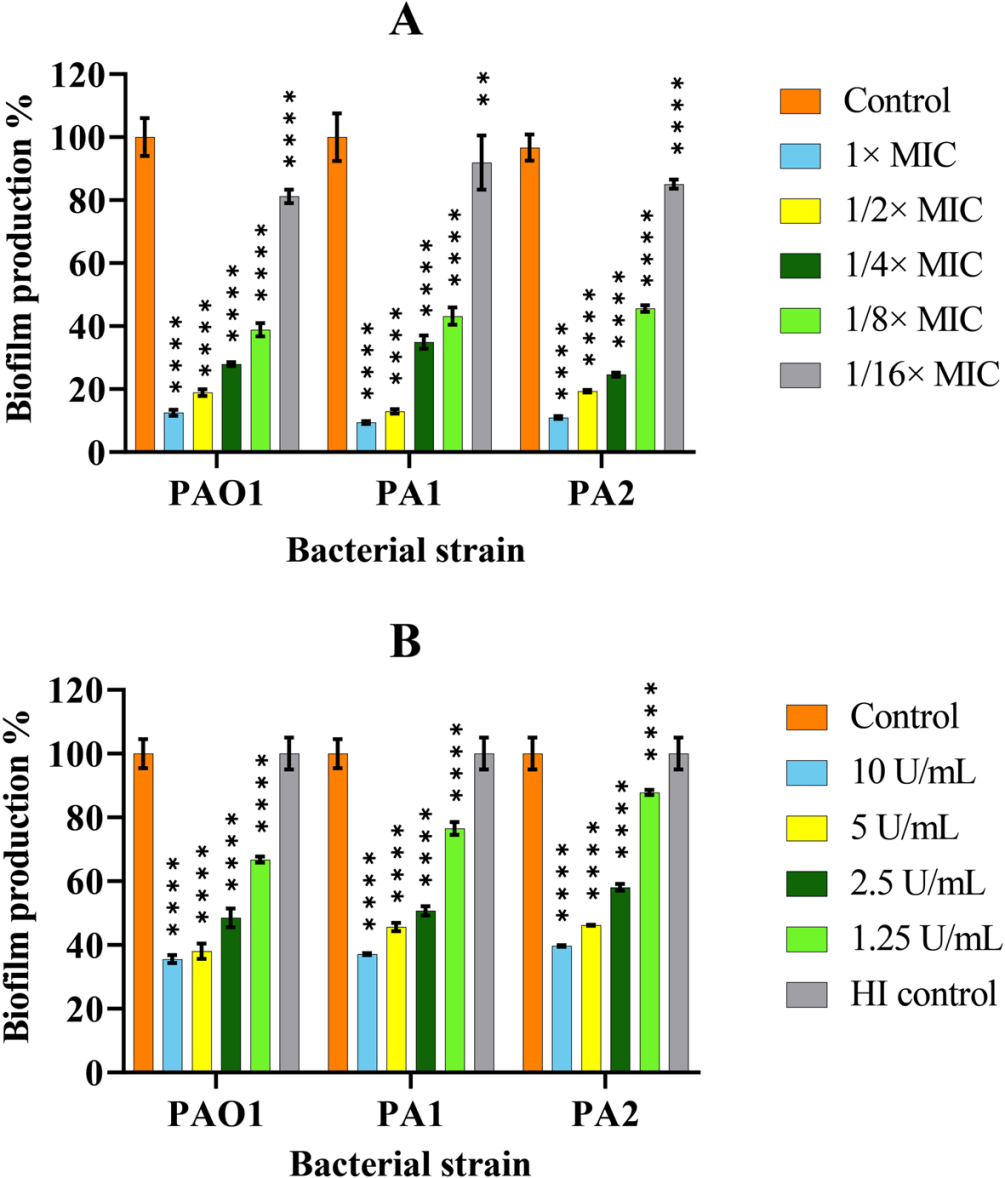
Reduction in the *P. aeruginosa* biofilm formation by PAO1, PA1, and PA2 with different concentrations of ceftazidime (**A**) and cellulase (**B**). The error bars indicate the standard deviations between strains. The microplates were incubated for 24 h at 37 °C. Data were normalized to the mean value of the control, which was set at 100%. The error bars indicate the standard deviations between bacteria. Results were expressed as percentage of biofilm biomass formed with respect to control. The statistical significance of the data was determined by an analysis of variance (ANOVA) test followed by the Tukey-Kramer multiple comparison test. Significance was accepted when the *P*-value was < 0.05 (****P < 0.0001, **P < 0.01). HI: Heat-inactivated; MIC: Minimum inhibitory concentration

The ability of ceftazidime and cellulase at different concentrations to inhibit biofilm formation was evaluated by CV staining after a 24 h-incubation of *P. aeruginosa* strains. As shown in Fig. 2, both antibiotic and enzyme significantly decreased biofilm formation in all strains in a dose-dependent manner compared to cells incubated in medium only (*P* < 0.0001). There was only moderate biofilm inhibition when all *P. aeruginosa* strains were incubated with ceftazidime at a concentration of 1/16× MIC (Fig. 2 A). Likewise, the 1.25 U/mL concentration of cellulase showed minor activity in the production of biofilm in all *P. aeruginosa* strains (Fig. 2B).

### Biofilm treatment with enzyme

The biofilm detachment activity of serial dilutions of cellulase against *P. aeruginosa* is shown in Table 2. Cellulase showed a significant detachment effect on biofilms at three concentrations of 10 U/mL, 5 U/mL, and 2.5 U/mL, with a biofilm reduction of 77.3-78.6%, 72.1-73.1%, and 78.3-78.4% in PAO1, PA1, and PA2, respectively, compared to the untreated control biofilms (*P* < 0.0001) (Data are also shown in supplementary Fig. 1). In addition, no reduction in biofilm biomass was observed with the HI enzyme.

**Table 2:** The effects of serial dilutions of cellulase on reduction of mature biofilms of *P. aeruginosa* strains

^a^Bacterial strain	% of biofilm forming reduction by cellulase concentration (U/mL) of										
	0.01	0.03	0.07	0.15	0.31	0.62	1.25	2.5	5	10	No/inactivated enzyme
PAO1	0.2	0.2	0.2	0.3	0.2	3	26.3	76.2	78.6	77.3	0
PA1	0.2	0.2	0.3	0.4	0.3	2.9	10.8	72.1	72.7	73.1	0
PA2	0.2	0.2	0.2	0.2	0.5	3.9	17.4	78.4	77.9	78.3	0

^a^ PAO1 is a laboratory reference strain of *P. aeruginosa* and PA1 and PA2 are two clinical isolates of *P. aeruginosa* that were recovered from the patients with burn wound infections

### MBECs of ceftazidime

The MBEC results for bacterial biofilm are listed in Table 1. Data indicated that all three isolates, which are ceftazidime-susceptible in the planktonic state, showed a dramatic increase in resistance to ceftazidime when grown in biofilms (*P* < 0.0001); the ceftazidime MBEC was 1024 to 4096-fold higher (range 2048 to 8192 µg/mL) compared with the MIC (range 2 to 4 µg/mL). Likewise, compared with the MBC value (range 4 to 8 µg/mL), the MBEC was 512 to 2048-fold higher (range 512 to 2048 µg/mL).

### Combination effect of cellulase and ceftazidime on biofilm formation

**Fig. 3:**
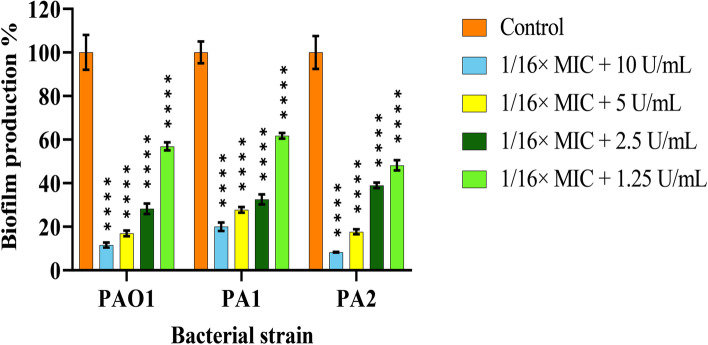
Reduction of *P. aeruginosa* biofilm formation by combination of ceftazidime (1/16× MIC) and different concentrations of cellulase in PAO1, PA1, and PA2. The error bars indicate the standard deviations between strains. The microplates were incubated for 24 h at 37 °C. Data were normalized to the mean value of the control, which was set at 100%. The error bars indicate the standard deviations between bacteria. Results were expressed as percentage of biofilm biomass formed with respect to control. The statistical significance of the data was determined by an analysis of variance (ANOVA) test followed by the Tukey-Kramer multiple comparison test. Significance was accepted when the *P*-value was < 0.05 (****P < 0.0001). MIC: Minimum inhibitory concentration

The sub-inhibitory concentration of 1/16× MIC of ceftazidime was chosen in combination with different concentrations of cellulase to assess the inhibitory effect of biofilm formation. Combination of the enzyme at all concentrations used with 1/16× MIC of antibiotic led to biofilm biomass reduction statistically higher than that caused by cellulase or ceftazidime used at the same concentrations alone (Fig. 3).

### The effect of cellulase on ceftazidime MBEC

The combination of cellulase at all concentrations of 2.5, 5, and 10 U/mL and ceftazidime significantly decreased the ceftazidime MBECs from 32- to 128-fold (*P* < 0.05) (Table 3). In particular, the enzyme significantly reduced the ceftazidime MBEC in the PAO1 strain up to 128 times.

**Table 3:** The combined effects of cellulase and ceftazidime on MBEC values of ceftazidime in *P. aeruginosa* strains

Bacteria	MBEC value (µg/mL) of		Fold reduction in ceftazidime MBEC in the presence of cellulase
	Ceftazidime	Ceftazidime + cellulase	
PAO1	2048	16	128
PA1	4096	64	64
PA2	8192	256	32

*MBEC* Minimum biofilm eradication concentration

## Discussion

Generally, biofilm-associated infections are complicated to successfully treat due to high resistance to antibiotics [28, 29]. To overcome biofilm-associated resistance, new therapeutic approaches have been of interest to the scientific community and are being evaluated. In recent years, enzymes have been considered as potential anti-biofilm agents. Different types of enzymes have been used as anti-biofilm agents in treating infections or wound debridement [30–34]. Several studies have also demonstrated the ability of exogenous glycoside hydrolases to inhibit biofilm formation and degrade preexisting biofilms [1, 17]. Baker et al. have recently shown that Pel- and Psl-specific glycoside hydrolases, PelA_h_ and PslG_h_, can inhibit the formation of and degrade biofilms of *P. aeruginosa in vitro* [35].

In this study, we investigated the *in vitro* activities of anti-pseudomonal antibiotic, ceftazidime, and the glycoside hydrolase enzyme, cellulase, alone and in combination against *P. aeruginosa* planktonic cells and biofilms. Results showed that cellulase had no bactericidal effect at the concentrations tested, consistent with those obtained in other studies that tested different enzymes [1, 35, 36]. Also, the destructive activity of various enzymes, including cellulase, on bacterial biofilms has been shown [1, 17, 37–39]. Two separate studies by Fleming et al. illustrated these results well. One study demonstrated that a dual-enzyme combination of α-amylase and cellulase don’t have any bactericidal activity but can significantly reduce the biofilm biomass of *Staphylococcus aureus* and *P. aeruginosa*, grown *in vitro* and *in vivo*, leading to the cell dispersal [1]. The second study showed that treatment of 48-hour-old *S. aureus* and *P. aeruginosa* mouse wound infections with 10% GH, α-amylase, and cellulase resulted in dispersal and significant septicemia, whereas pre-treating with 10% GH before inoculation did not cause an increase in septicemia, indicating that the effect of GH on bacteremia is directly on the biofilm [40]. Nagraj et al. showed an enzyme complex containing amylase, cellulase, and protease degraded biofilm of *P. aeruginosa* as high as 88.76% within one h of incubation [17]. Furthermore, a recent study demonstrated that the use of cellulase in multi-well plate biofilm dispersal assays leads to increased percent dispersal of *Entrococcus faecalis* [41]. Collectively, the biofilm degradation of bacteria by glycoside hydrolases, such as cellulase may be due to the hydrolysis of polysaccharides and disruption of the biofilm architecture that increased dispersal of planktonic cells. As a result, an increased rate of planktonic cell killing occurs by both the immune system and antimicrobial agents [6, 42] due partly to the higher metabolic rates and better cell surface access to free-floating cells.

When we examined the MIC values of ceftazidime against planktonic *P. aeruginosa* cells, they ranged between 2 and 4 µg/mL. When we considered the anti-biofilm activities of this antibiotic, MBEC values ranged between 2084 and 8192 µg/mL. According to these results, the MBEC/MIC ratio of this active antibiotic was found to be within the range of 1024- to 4096-fold. These results are similar to those obtained by others [43, 44].

The bacterial adhesion to surfaces is an essential step for biofilm formation. Many continuing studies target the inhibition of this critical step. In this study, we investigated the inhibition of bacterial attachment to the surfaces and the inhibition of biofilm production by MIC or subMIC values of ceftazidime or different concentrations of cellulase. Ceftazidime inhibited the attachment of PAO1 strain in a concentration- and time-dependent manner (*P* < 0.0001). Similar results were reported by Dosler et al. [43], where different antimicrobial agents, including ceftazidime at 1/10× MIC inhibited the biofilm attachment in a time-dependent fashion. The time-dependent killing of ceftazidime on planktonic *P. aeruginosa* cells, as demonstrated by Hengzhuang et al. [45], might explain no inhibitory effect of antibiotic on biofilm attachment observed in two clinical isolates. Neither of the tested concentrations of cellulase inhibited biofilm attachment in all bacteria, in accordance with results obtained by Cardeiro and coworkers [46]. Their results showed that subtilisin A reduced the attachment of *P. aeruginosa* by 44% onto a copolymer film, but cellulase had no significant effect, suggesting a more prominent role of proteins than polysaccharides in the initial attachment of *P. aeruginosa* to surfaces [46].

Our results also showed that ceftazidime and cellulase significantly inhibit biofilm formation in a concentration-manner, especially at 1× or 1/2× MIC (*P* < 0.0001). Similarly, Otani et al. observed a dose-dependent manner of reduction in biofilm formation by PAO1 strain with different concentrations (0, 1/16, 1/8, 1/4, 1/2, 1× MIC) of ceftazidime [47]. Given the bactericidal mechanism of ceftazidime, this anti-biofilm activity can be attributed to reducing bacterial density in a microbial population. Besides, downregulation of the *pelA* and *pslA* genes by subMIC ceftazidime has been shown by Otani et al. [47], suggesting a mechanism responsible for inhibited biofilm formation in *P. aeruginosa*. Likewise, significant reduction of biofilm formation at different cellulase concentrations indicates that enzyme targets polysaccharides playing during biofilm development and maturation in *P. aeruginosa*.

Moreover, our data demonstrated that ceftazdidme/cellulase combination significantly decreased biofilm formation compared to when agents were used at the same concentrations alone (*P* < 0.0001). The increased susceptibility of *P. aeruginosa* to various antibiotics in combination with PelA_h_ and PslG_h_ enzymes has been reported in two individual studies by Baker and colleagues [35, 48]. They demonstrated that treatment of PAO1 biofilms *in vitro* with these GH enzymes improves the efficacy of tobramycin, polymyxin, colistin, and neomycin, leading to nearly 1 log greater bacterial killing than antibiotic treatment alone [48]. Moreover, PelA_h_ and PslG_h_ treatment [35] and PslG_h_ treatment [48] improves colistin and tobramycin penetration into the *P. aeruginosa* biofilm, leading to reduced viable bacterial counts in culture and infected wounds, respectively. In the present study, the cellulase was also highly efficient with ceftazidime and reduced the MBEC 32 to 128 times. Several studies found the ability of different enzymes to decrease the MBEC of antibiotics, including ceftazidime [36, 38, 43, 49]. The synergistic effect achieved by using enzyme/antibiotic combination can rapidly enhance anti-biofilm activity and help prevent or delay the emergence of resistance.

Our data showed the anti-biofilm activity of cellulase, separately and in combination with ceftazidime, against *P. aeruginosa*. So, further studies are recommended to perform on it. The cytotoxic effect of the enzyme must be evaluated. Given the polymicrobial nature of biofilms, it is proposed to investigate the cellulase’s efficacy on the destruction of mixed-species biofilms. It must be addressed whether the enzyme increases the effectiveness of all antibiotics or just certain classes. Moreover, the effects of the enzyme on immune system response to clear infections should be examined. Thus, these are just some of the questions needed to be answered before clinical applications of cellulase can be explored.

## Conclusions

Considering the anti-biofilm properties of glycoside hydrolase cellulase in this study, including significant degradation of *P. aeruginosa* biofilm and a significant decrease in the ceftazidime MBEC, it appears to be a promising candidate, either as a single agent or as in combination with antibiotics for disturbing/dispersing biofilm and making highly resistant wound infections susceptible to traditional treatments.

## Supplementary Information

**Additional file 1.** The effects of selected concentrations of cellulase (0.01-10 U/mL) against biofilm embedded *P. aeruginosa* isolates and ATCC PAO1 strain.

## Data Availability

The datasets analyzed during the current study are available from the corresponding author on reasonable request.

## Abbreviations

CAMHB: Cation-adjusted Mueller-Hinton broth
CLSI: Clinical and Laboratory Standards Institute
CV: Crystal violet
EPSs: Extracellular polymeric substances
GH: Glycoside hydrolase
HI: Heat-inactivated
MBC: Minimum bactericidal concentration
MBEC: Minimum biofilm eradication concentration
MHB: Mueller–Hinton broth
MIC: Minimum inhibitory concentration
NIH: National Institutes of Health
TSB: Tryptic soy broth
