# Supplementary figures and images for: In vitro activities of cellulase and ceftazidime, alone and in combination against Pseudomonas aeruginosa biofilms
Source: BMC Microbiol. 2021 Dec 16;21:347. doi: 10.1186/s12866-021-02411-y (PMC8675527; doi:10.1186/s12866-021-02411-y)

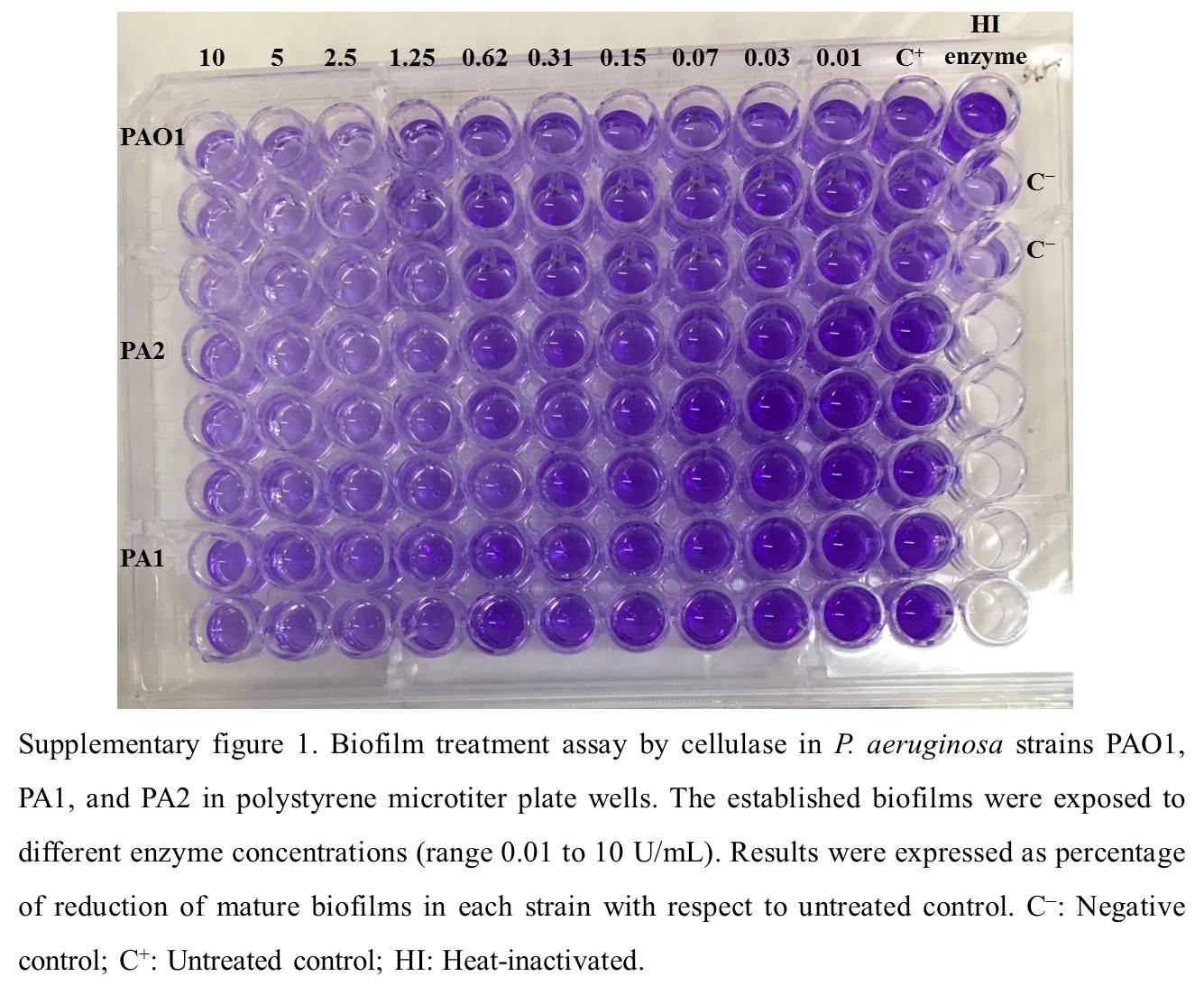

Supplement: Supplementary file 1 — Additional file 1. The effects of selected concentrations of cellulase (0.01-10 U/mL) against biofilm embedded P. aeruginosa isolates and ATCC PAO1 strain. [file 12866_2021_2411_MOESM1_ESM.jpg]
